# Effects of Land Reclamation on Soil Bacterial Community and Potential Functions in Bauxite Mining Area

**DOI:** 10.3390/ijerph192416921

**Published:** 2022-12-16

**Authors:** Xuesong Li, Zhenjiang Jin, Liyuan Xiong, Lingchen Tong, Hongying Zhu, Xiaowen Zhang, Guangfa Qin

**Affiliations:** 1 College of Environmental Science and Engineering, Guilin University of Technology, Guilin 541004, China; 2 Collaborative Innovation Center for Water Pollution Control and Water Safety in Karst Area, Guilin University of Technology, Guilin 541004, China; 3 Guangxi Key Laboratory of Environmental Pollution Control Theory and Technology, Guilin University of Technology, Guilin 541004, China

**Keywords:** bauxite mine, soil reclamation, high-throughput sequencing, bacterial community, functional taxa, co-occurrence networks

## Abstract

Studying the characteristics of microorganisms in mine reclamation sites can provide a scientific reference basis for mine land reclamation. Soils in the plough layer (0–20 cm) of the bauxite mine plots in Pingguo city, Guangxi Zhuang Autonomous Region, China, with different reclamation years were used as the research objects. The community structure of soil bacteria was analyzed with high-throughput sequencing technology. The results show the following: (1) Reclamation significantly increased the contents of soil nutrients (*p* < 0.05). (2) The relative abundances of Proteobacteria were high (22.90~41.56%) in all plots, and reclamation significantly reduced the relative abundances of Firmicutes (3.42–10.77%) compared to that in the control plot (24.74%) (*p* < 0.05). The relative abundances of α-proteobacteria generally increased while the reclamation year increased. The relative abundances of α-proteobacteria and γ-proteobacteria showed significant positive correlations with soil carbon, nitrogen, and phosphorus nutrients (*p* < 0.01). The relative abundance of Acidobacteria Group 6 showed significant positive correlations with soil exchangeable Ca and Mg (*p* < 0.01). (3) Bacterial co-occurrence network showed more *Copresence* interactions in all plots (50.81–58.39%). The reclaimed plots had more nodes, higher modularity, and longer characteristic path length than the control plot, and the keystone taxa changed in different plots. (4) The chemoheterotrophy and aerobic chemoheterotrophy were the most abundant functional groups in all plots (35.66–48.26%), while reclamation reduced the relative abundance of fermentation groups (1.75–11.21%). The above findings indicated that reclamation improved soil nutrients, changed the bacterial community structure and potential functions, and accelerated the microbial stabilization of the reclaimed soil.

## 1. Introduction

Bauxite, a typical exogenous mineral resource, is formed by intense chemical weathering in humid and hot areas [1]. Bauxite deposits formed on the paleokarstic surfaces of carbonates, which are considered carbonate bauxite [2,3], are often located 1–2 m below the surface, and therefore they are usually mined by opencast mining (strip mining) [3]. The large-scale opencast mining process directly destroys surface rock layers and vegetation [4], leading to changes in the physical structure and loss of soil organic matter [5], drastically reducing the soil nutrient contents [6] and causing serious ecological damage [7]. At the same time, the bauxite mining process occupies a large number of cultivated lands. The ecological restoration of bauxite mining areas, therefore, is particularly important in karst areas where the quantity and quality of cultivated lands are small and poor.

Reclamation in mining areas is one of the measures to promote the recovery of soil productivity and to guarantee the sustainable and effective use of land resources [8]. The bauxite mining process usually retains top soils for reclamation and then plants fast-growing local dominant plants, nitrogen-fixing legumes, or economic crops for restoration. During reclamation, the growth of plant roots can improve soil physicochemical properties, enhance the accumulation of soil nutrients [9,10], and promote the recovery of soil microorganisms [9]. In return, the accumulation of nutrients can promote the growth and development of reclamation plants [11] as well as increase the productivity of reclaimed soils. Soil microorganisms, which have a variety of physiological and biochemical functions, are the main drivers of biogeochemical cycles [12]. On the one hand, the reclamation process can increase soil bacterial abundance and diversity [13], change soil bacterial community composition [10], and improve bacterial community stability. On the other hand, bacteria respond sensitively to changes in their survival environment [14]. They can participate in organic matter decomposition, humus formation, and material cycling [15] and promote the formation of soil structure [16]. They can also promote hydrolysis reactions to release phosphorus, potassium, and other nutrients from soil minerals to enhance the soil weathering process through the production of organic acids [17], which helps increase the fertility of reclaimed soil. Therefore, the bacteria in reclaimed soils may serve as an important criterion to assess the success of reclamation [18].

The bauxite mining process caused a decrease in nutrient content such as soil organic carbon (SOC) [5,19], an increase in bulk density [20,21], and a decrease in bacterial abundance and diversity [22]. The process significantly changed the soil bacterial community structure. However, with the increase in reclamation years, soil carbon, nitrogen, and phosphorus nutrients gradually accumulated [23], physical structure improved [24], and soil bacterial community structure gradually recovered to be closer to that of undisturbed soil [22,25]. Soil pH and SOC were the most important physicochemical properties affecting the bacterial community [25]. These studies above indicate that current research on reclaimed soils in bauxite mines has focused on the effects of reclamation time on soil physicochemical properties, nutrient contents, and bacteria community structure. However, studies on the changes in potential functional taxa of bacteria and the interactions among bacteria in reclaimed soils of bauxite mines have not been reported. Therefore, soils in the plough layer of the bauxite mine sites located in Pingguo city, Guangxi Zhuang Autonomous Region, China, with different reclamation years were used as the research objects, and the following studies were conducted: (1) the effects of reclamation on the soil physicochemical properties; (2) the effects of reclamation on the structure of soil bacterial communities; (3) the effects of reclamation on the interactions of soil bacteria and their keystone taxa in the co-occurrence network; and (4) the effects of reclamation on the potential functional groups of soil bacteria. This study aims to elucidate the effects of reclamation on soil microbial community structure and potential functions and to provide a scientific basis for accelerating the ecological recovery of reclaimed soils in bauxite mines.

## 2. Materials and Methods

### 2.1. Overview of the Study Area

**Figure 1:**
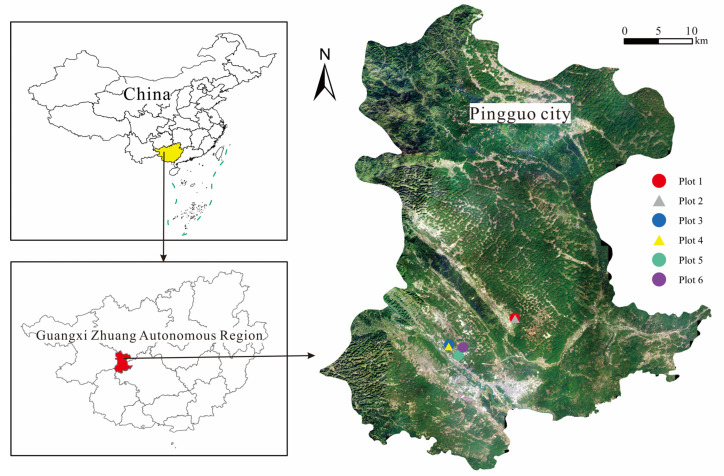
Location of the study area.

The study area (Figure 1) is located in Pingguo city, Guangxi Zhuang Autonomous Region, China (107°26′05″–107°39′22″ E, 23°19′58″–23°28′55″ N). The area has an average annual temperature of 21.7 °C and an average annual rainfall of 1309.8 mm, which is a typical subtropical hot and humid climate. Soluble carbonate rocks are widely distributed in the bauxite mining area of Pingguo city, and tectonic movements and strong karst action have formed karst landforms of different forms, which promote the formation of karst-accumulation-type bauxite [26]. The mine area is mainly purple-red colluvial clay [27], and the dominant vegetation is *Miscanthus floridulus*, *Neyraudia renaudiana*, and *Bidens pilosa*. According to the field survey and literature reports, the reclamation project of the Pingguo bauxite mining area mainly backfills the pits, holes, and low areas of the reclamation sites with clint and earth-stone works for site leveling [27], then fills the surface layer with the base soil of about 0.5 m thickness, which comes from the filter cake of the washed tailing mud after pressing and filtering. The base soil is used as the water retention layer of the base of reclamation site for leveling again. The upper layer of the base soil is filled with about 0.5 m of cultivated soil, which mainly comes from the stripping soil during mining process [28].

### 2.2. Soil Sampling and Pre-Treatment

Eighteen soil samples (6 plots × 3 replicates) of plough layer soil (0–20 cm) were collected in November 2020 in the bauxite area to represent different reclamation years. Samples collected from the leveled plot were identified as control soil, and those with reclaimed plants were identified as the reclaimed soil (details of the plots are shown in Table 1). Plant roots, small stones, and other debris were removed from the collected samples after being transferred to the laboratory. Equal amounts of samples from each plot were mixed thoroughly and divided into two parts: one part was stored at −80 °C for subsequent analysis of microbial community structure, and the other part was air-dried and ground through 10-mesh, 60-mesh, and 100-mesh sieves, respectively, and stored for the determination of physicochemical properties.

### 2.3. DNA Extraction, Sequencing, and Sequencing Data Processing

Total genome DNA from samples was extracted using the CTAB method. DNA concentration and purity were monitored on 1% agarose gels. According to the concentration, DNA was diluted to 1 ng/µL using sterile water. 16S rRNA genes of distinct regions (16S V4) were amplified using specific primers (515F (GTGCCAGCMGCCGCGGTAA) and 806R (GGACTACNNGGGTATCTAAT)) with the barcode. All PCR reactions were carried out with 15 µL of Phusion^®^ High-Fidelity PCR Master Mix (New England Biolabs, Ipswich, MA, USA), 2 µM of forward and reverse primers, and about 10 ng template DNA. Thermal cycling consisted of initial denaturation at 98 °C for 1 min, followed by 30 cycles of denaturation at 98 °C for 10 s, annealing at 50 °C for 30 s, and elongation at 72 °C for 30 s, with a final extension at 72 for 5 min. The same volume of 1XTAE buffer was mixed with PCR products, and electrophoresis was performed on 2% agarose gel for detection. PCR products were mixed in equidensity ratios. Then, the mixture of PCR products was purified with Qiagen Gel Extraction Kit (Qiagen, Hilden, Germany). The library was sequenced on an Illumina NovaSeq platform (PE 250) by Wekemo Tech Group Co., Ltd., Shenzhen, China.

Demultiplexed sequences from each sample were quality filtered and trimmed, de-noised, and merged, and then the chimeric sequences were identified and removed using the QIIME 2 dada 2 plugin to obtain the feature table of amplicon sequence variant (ASV) [29]. The QIIME 2 feature-classifier plugin was then used to align ASV sequences to a pre-trained GREENGENES 13_8 99% database to generate the taxonomy table [30].

The α-diversity indices (including Chao1, ACE, Shannon, and Simpson indices) were used to characterize the abundance and diversity of the bacterial communities. Among them, Chao1 and ACE indices were used to characterize microbial abundance, and Shannon and Simpson indices were used to characterize microbial community diversity.

### 2.4. Soil Physicochemical Properties Analysis

Soil physicochemical properties were analyzed as described in Soil Agricultural Chemical Analysis [31]. Briefly, pH was determined with a precision pH meter (soil/water ratio = 1:2.5, *m*/*v*). Soil organic carbon (SOC) was determined by the concentrated sulfuric acid-potassium dichromate external heating method. Dissolved organic carbon (DOC) was determined with a total organic carbon analyzer (multi N/C 3100, Analytik Jena) after leaching with ultrapure water (soil/water ratio = 1:10, *m*/*v*). Total nitrogen (TN) was determined with an elemental analyzer. Alkaline hydrolytic nitrogen (AN) was determined by the alkaline diffusion method; Ammonium nitrogen (NH_4_^+^-N) and nitrate nitrogen (NO_3_^−^-N) were extracted with 2 M KCl, and the concentration was determined by UV spectrophotometric method [32]. Total phosphorus (TP) was determined by the acid solubilization-molybdenum antimony resistance method. Olsen-P (AP) was extracted with 0.5 M NaHCO_3_, and the concentration was determined by UV spectrophotometric method [33]. Cation exchange capacity (CEC) was determined by the hexaamminecobalt trichloride solution-spectrophotometric method (HJ 889-2017). The concentrations of exchangeable calcium (Ca^2+^) and exchangeable magnesium (Mg^2+^) were determined by ICP-OES (Perkin Elmer, Waltham, MA, USA) [34].

### 2.5. Data Analysis

The α-diversity indices and the analysis of similarity (ANOSIM) were calculated and performed in R language. Analysis of variance (ANOVA) and Pearson’s correlation analysis was performed with SPSS 26.0 software (IBM, Armonk, NY, USA). Principal component analysis (PCoA) of the bacterial communities was performed with Canoco 5 software (Microcomputer Power, Ithaca, NY, USA). Histograms and box plots were plotted with Origin 2018. Bacterial co-occurrence networks were constructed with soil bacterial ASVs (relative abundance > 0.1%) in each plot by the method of the CoNet plugin in Cytoscape 3.8.2. Briefly, pairwise associations among bacterial ASVs were calculated by Pearson correlation, Spearman correlation, Bray–Curtis dissimilarity, and Kullback–Leibler dissimilarity [35]. The initial network edges were retained only if they were supported by at least two relevant methods. Correlations of ASVs with *p* < 0.05 were retained (after the randomization routine of Brown and bootstrap) to construct the correlation network. Network Analyzer tool was used for network topology parameter calculation [36], and Gephi 0.9.2 was used for visualization. The ASVs with Closeness Centrality > 0.12, Betweenness Centrality < 0.10, and Degree ranking top 5 were identified as keystone taxa [37]. Functional Annotation of Prokaryotic Taxa (FAPROTAX) based on bacterial abundance and annotation information was performed with online software (http://www.ehbio.com, accessed on 31 March 2022) [38].

## 3. Results

### 3.1. Characteristics of Soil Physicochemical Properties in Different Plots

According to the results of soil physicochemical properties measured in different plots (Table 2), reclamation significantly increased the contents of SOC, DOC, TN, AN, NH_4_^+^-N, and Mg^2+^ compared to Plot 1 (*p* < 0.05). The contents of SOC, DOC, and TP in reclaimed soils showed a general increasing trend with the increase in reclamation years. The carbon, nitrogen, and phosphorus nutrients were significantly higher in Plot 6 (*p* < 0.05), which had been reclaimed for 12 years, than those in the rest of the plots. Compared to the second national soil census nutrient classification standard of China (Table S1), the contents of SOC, AN, TP, and AP showed low concentration in Plot 1 to Plot 3 (SOC < 10 g·kg^−1^, AN < 60 mg·kg^−1^, TP < 0.5 g·kg^−1^, and AP < 5 mg·kg^−1^), while the contents of SOC, TN, TP, and AP were high (30 g·kg^−1^ < SOC < 40 g·kg^−1^, TN > 2 g·kg^−1^, TP >2 g·kg^−1^, and AP > 40 mg·kg^−1^) in Plot 6. The above results indicate that after the reclamation measures, the soil physicochemical properties have been remarkably improved.

### 3.2. Bacterial Diversity and Community Composition of Soils in Different Plots

#### 3.2.1. Bacterial Diversity in Different Plots

**Figure 2:**
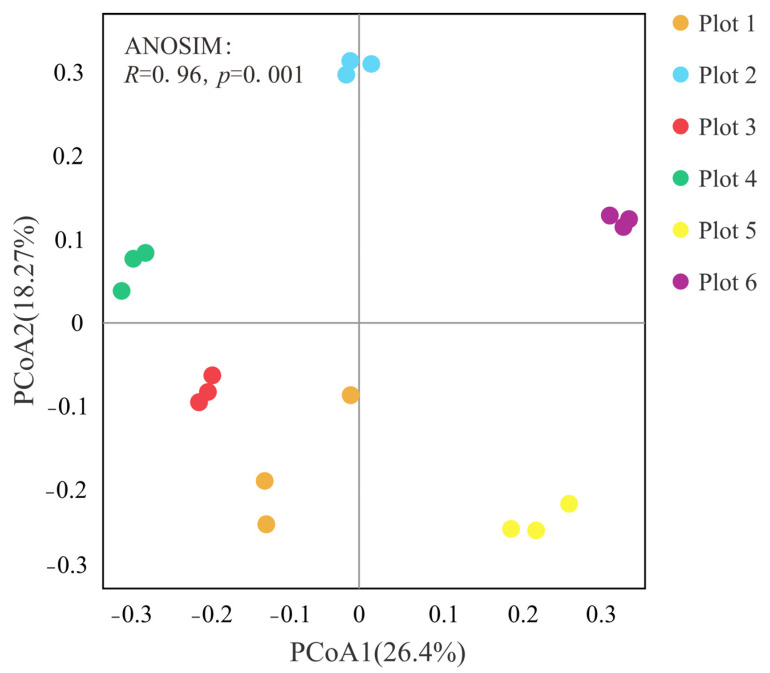
Principal component analysis (PCoA) of bacterial community structure.

PCoA and ANOSIM analysis based on the Bray–Curtis distance of the bacterial community at the level of ASVs estimated the β-diversity of soil bacterial community in different plots (Figure 2). It can be seen that axis 1 and axis 2 explained 26.40% and 18.27% of the variance, respectively, with a total variability of 44.67%. Moreover, the ANOSIM analysis showed significant variation among soil bacterial community structures (*R* = 0.96, *p* = 0.001) in different plots.

The α-diversity indices of soil bacteria in different plots were calculated and statistically analyzed (Table 3). It was found that the Chao1 and ACE indices of soil bacteria in Plot 4 and the Shannon and Simpson indices in Plots 5 and 6 were significantly higher than those in Plot 2 (*p* < 0.05). None of the four indices showed an increasing pattern with the increase in reclamation years.

#### 3.2.2. Bacterial Community Composition of Reclaimed Plots

**Figure 3:**
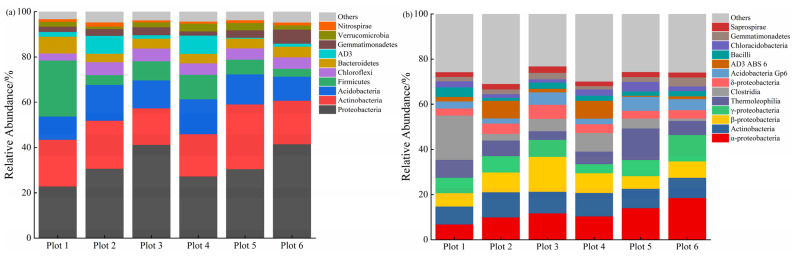
Bacterial community composition at the level of phylum (**a**) and class (**b**) in different plots. The phyla/classes with relative abundance greater than 1%/2% were defined as dominant phyla/classes, and sequences not classified to any taxonomy and phylogenetic groups accounting for ≤1%/2% of all classified sequences are summarized in the group “Others”.

The bacterial distribution was different in different plots (Figure 3). The categorization of ASVs showed 10 dominant phyla (Figure 3a), and their relative abundance ranged from 95.30% to 96.83% of the total bacteria. Proteobacteria was the most abundant phylum (32.40%). The relative abundance of Firmicutes in Plot 1 (24.74%) was significantly higher than that in the rest of the plots (3.42~10.77%) (*p* < 0.05), and the relative abundance of Bacteroidetes (7.45%) was significantly higher than that in the rest of the plots (3.72~4.34%) except Plot 6 (*p* < 0.05).

At the class level, 13 dominant classes were classified (Figure 3b), and their relative abundances ranged from 69.11% to 76.88% of the total bacteria. The relative abundance of α-proteobacteria roughly showed an increasing trend with the increase in reclamation years. Statistical analysis showed that the relative abundance of Clostridia in Plot 1 (19.69%) was significantly higher than that in the rest of the plots (1.01~8.21%) (*p* < 0.05). The relative abundance of β-proteobacteria in Plot 3 (15.48%) was significantly higher than that in the rest of the plots (5.61~8.77%) (*p* < 0.05). The relative abundance of γ-proteobacteria in Plot 6 was significantly (11.73%) the highest (*p* < 0.05). The relative abundances of Acidobacteria Group 6 (Gp6) in Plot 3, Plot 5, and Plot 6 were significantly higher than those in the rest of the plots (4.03~7.51%) (*p* < 0.05).

### 3.3. Analysis of Soil Bacterial Co-Occurrence Networks in Different Plots

**Figure 4:**
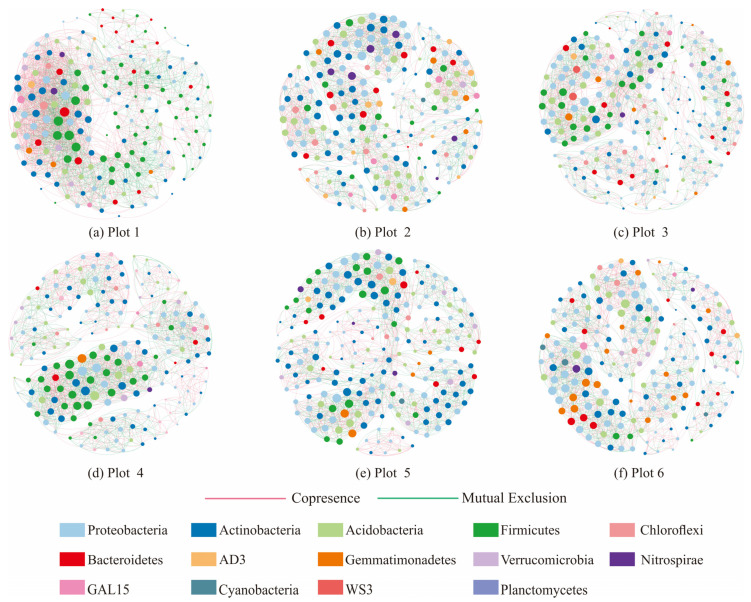
(**a**–**f**) Co-occurrence networks of bacteria at the ASV level in different plots.

There are complex interactions among soil microorganisms. These interactions can be integrated by employing co-occurrence networks to be visualized [39]. Therefore, in this study, we constructed a bacterial network for each plot (Figure 4) and described the complex interactions among ASVs by the calculated results of network topological characteristics (Table 4) [40]. The results show that *Copresence* interactions among soil bacteria were dominant in all plots. Some of the topological characteristic indices of the bacterial network in Plot 1 were the lowest, such as the number of nodes, modularity, characteristic path length, clustering coefficient, and network density, while the number of edges, number of *Copresence* edges, average number of neighbors, network density, and network central potential were all the highest. The results indicate that the bacterial network in the control plot was more complex and networks in the reclaimed soils were more stable.

Information on the keystone taxa of soil bacteria in each plot (Table 5) showed that the bacterial network in Plot 1 was mainly influenced by Firmicutes and Bacteroidetes. The bacterial network in Plot 2 was mainly influenced by Proteobacteria, Acidobacteria, and Actinobacteria. The bacterial network in Plot 3 was mainly influenced by Firmicutes, Bacteria WS3, and Actinobacteria. The bacterial network in Plot 4 was mainly influenced by Firmicutes, Acidobacteria, Proteobacteria, and Actinobacteria. The bacterial network in Plot 5 was mainly influenced by Gemmatimonadetes, Proteobacteria, and Acidobacteria. The bacterial network in Plot 6 was mainly influenced by Proteobacteria, Acidobacteria, and Gemmatimonadetes. The results indicate that the keystone taxa of bacterial co-occurrence networks of different plots changed. In addition, some bacteria with low abundance were identified as keystone species, indicating that keystone taxa might be independent of the relative abundances.

### 3.4. Correlations between Soil Bacterial Community Structure and Soil Physicochemical Properties

**Figure 5:**
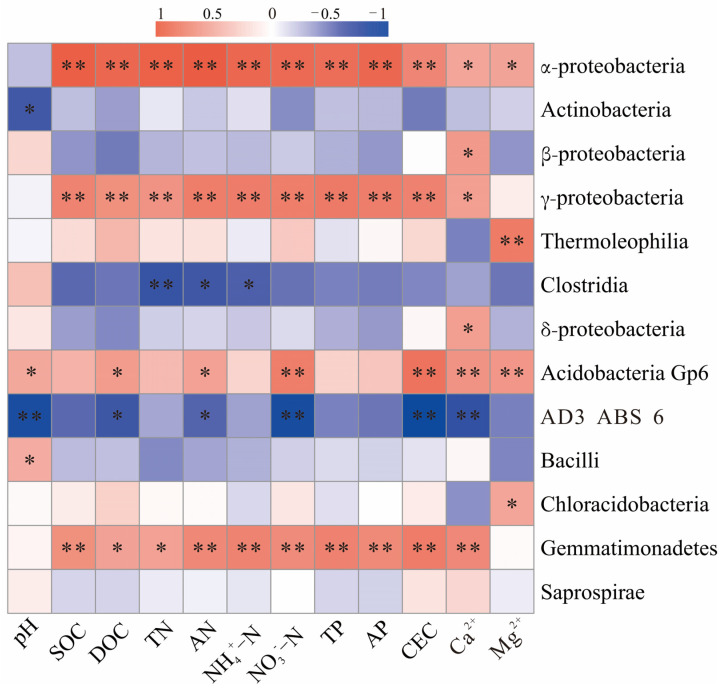
Correlations of dominant classes and soil physicochemical properties. * *p* < 0.05 (Pearson correlation, two-tailed), ** *p* < 0.01 (Pearson correlation, two-tailed).

Correlation analysis of soil bacterial dominant classes and physicochemical properties (Figure 5) showed that the relative abundances of both α-proteobacteria and γ-proteobacteria were highly significantly positively correlated with SOC, DOC, TN, AN, NH_4_^+^-N, NO_3_^−^-N, TP, AP, and CEC (*p* < 0.01). The relative abundance of Clostridia was highly significantly negatively correlated with TN (*p* < 0.01). The relative abundance of Acidobacteria Gp6 was significantly positively correlated with pH, DOC, and AN (*p* < 0.05) and highly significantly positively correlated with CEC, Ca^2+^, and Mg^2+^ (*p* < 0.01).

### 3.5. Functional Prediction of Soil Bacteria in Different Plots

**Figure 6:**
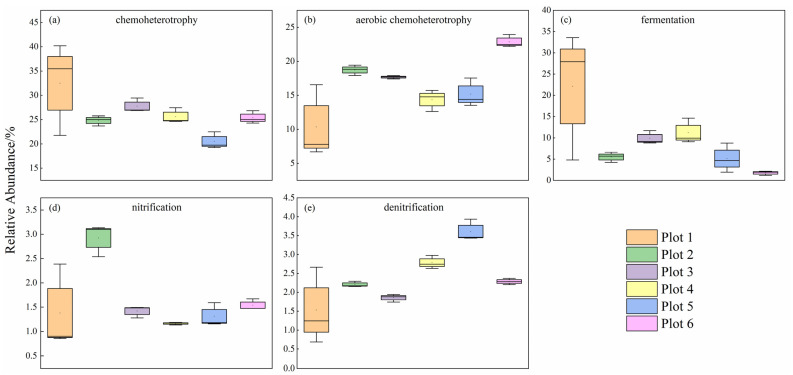
Changes in soil bacterial (**a**–**e**) potential functional groups in different plots. The range of the box is the mean ± SD, the vertical line shows the 10th to 90th percentile, and the horizontal black line in the box is the median values.

In this study, a total of 66 effective bacterial functional groups were predicted by the method of FAPROTAX. Five bacterial ecological functions related to carbon and nitrogen cycles were selected (Figure 6), namely chemoheterotrophy, aerobic chemoheterotrophy, fermentation, nitrification, and denitrification, which accounted for 45.69% to 67.83% of the total abundance of the predicted functional groups. The results show that the main bacterial functional taxa were chemoheterotrophy and aerobic chemoheterotrophy bacterial functional groups with a sum of relative abundance ranging from 35.66% to 48.26%. The relative abundance of fermentation bacteria in Plot 1 (22.09%) was significantly the highest (*p* < 0.05). The abundance of nitrification bacteria in Plot 2 (2.92%) was significantly the highest (*p* < 0.05), and the abundance of denitrification bacteria in Plot 5 (3.61%) was significantly the highest (*p* < 0.05).

## 4. Discussion

### 4.1. The Effects of Reclamation on Soil Nutrient Enhancement

In this study, all five plants significantly increased the contents of SOC, DOC, TN, AN, and NH_4_^+^-N in reclaimed plots, and the condition of soil nutrients improved with the reclamation year increase, indicating that reclamation could significantly enhance the nutrients of reclaimed soils. Mainly because the root exudates and the litters of reclaimed plants contain large amounts of C and N compounds such as polysaccharides, organic acids, and amino acids, which can increase soil nutrient accumulation [41]. At the same time, as the reclamation year increased, soil fertility increased under the action of weathering, precipitation, and other factors, and the nutrients increased as well. The contents of TN and NH_4_^+^-N in Plot 2 with *Glycine max* planted for 1 year and Plot 3 with *Leucaena leucocephala* planted for 4 years were comparable to those in Plot 4 with *Neyraudia reynaudiana* planted for 7 years, indicating that the soil nutrient condition was mainly limited by nitrogen at the early stage of succession [42], and legumes could rapidly increase soil nitrogen to accelerate the reclamation process. In contrast, *Neyraudia reynaudiana* planted in Plot 4 grew densely with a large amount of above-ground biomass and a large amount of litters. Part of the litters were input into the soil in the form of humic acid and humin [43] to exist stably by fermentation and decomposition, which, together with the longer reclamation years, resulted in more organic matter accumulation compared to Plot 2 and Plot 3. The decomposition process of organic fertilizers after application produced organic acids, which could promote the mineralization process and increase the effectiveness of soil nutrients [44]. The combined application of organic and inorganic fertilizers could also reduce the runoff loss of soil nitrogen [45]. Plot 5 and Plot 6 had been reclaimed for more than 10 years with more fertilizer inputs; therefore, soil nutrients in Plot 5 and Plot 6 showed the most significant enhancement.

### 4.2. Driving Effects of Reclamation on Changes in Soil Bacterial Community Structure

Soil microorganisms are extremely sensitive to environmental change. They can provide in-time responses to the recovery of soils in reclamation sites. Therefore, they might be important early indicators of land reclamation and ecological restoration in mining areas [18]. In this study, there were significant differences in bacterial community structure among reclaimed plots (*R* = 0.96, *p* = 0.001), and the long-term reclamation process (reclamation time >10 years) significantly increased the bacterial abundance and diversity in reclaimed soils. On the one hand, the reason for the changes in bacterial community structure may be due to the screening effect of root tissue abscission and exudates of different reclamation plants on soil bacterial taxa [46]. On the other hand, it may also be caused by complex and variable field conditions and different human management practices (fertilization, tillage, etc.). In addition, the availability of soil nutrients and the ability of bacteria to utilize soil nutrients under different reclamation conditions may also drive the changes in bacterial community structure [25].

Proteobacteria showed high relative abundances in all plots in this study (22.90~41.56%). They are able to use unstable carbon [47] and are more likely to maintain rapid and dominant growth in nutrient-rich soils, which might be due to their wide ecological niche with morphological, physiological, and metabolic diversity [13]. The α-proteobacteria, β-proteobacteria, γ-proteobacteria, and δ-proteobacteria belonging to Proteobacteria were all dominant classes, among which, the relative abundance of α-proteobacteria showed an increasing trend (6.91~18.58%) with the increase in reclamation years, which might be related to the increase in soil nutrients in reclaimed soil. It has been reported that soils with high contents of nutrient status are rich in α-proteobacteria [48], and the natural restoration process increases the abundance of α-proteobacteria [49]. The above two studies also corroborate our results. The α-proteobacteria could be further classified into three dominant families (relative abundance >1%) of Hyphomicrobiaceae, Rhodospirillaceae, and Sphingomonadaceae (Figure S1), among which the relative abundances of the first two families (1.84~4.98% and 1.03~3.68%) showed increasing trends with the increase in reclamation years, and they all showed highly significant positive correlations with SOC, DOC, TN, AN, NO_3_^−^-N, and AP (*p* < 0.01) (Figure S2). The family of Rhodospirillaceae, which is highly associated with the nitrogen fixation and nitrate reduction process [50], plays an important role in the soil nitrogen cycle. Sphingomonadaceae members secrete sugary nutrients in the rhizosphere to help against plant pathogens [51] and can metabolize a variety of carbon compounds [50], indicating their functions to maintain the carbon cycle and promote plant growth in the soil of mine reclamation sites. In addition, some keystone species (ASV 149, ASV 183) in Plot 1, Plot 4, and Plot 6 belonged to Rhodospirillaceae and Hyphomicrobiaceae. Therefore, it was clear that α-proteobacteria played an important role in the ecological restoration of reclaimed soils.

Actinobacteria had high abundances (16.18~28.63%) in all plots in this study, mainly due to their ability to utilize persistent and complex substrates in nutrient-limited soils, which could be beneficial to facilitating the mineralization of organic matter. The relative abundances of Firmicutes and Bacteroidetes were both higher in the control plot, while they both decreased, however, in varying degrees with the reclamation year increase. Most members of Firmicutes are pioneer species in reclaimed soil because they have special physiological structures such as spores [25], which can adapt to disadvantageous conditions such as the shortage of nutrients and water. Once environmental conditions ameliorate, they can quickly recover and participate in the soil biogeochemical cycle process, speeding up the recovery of soil quality in reclamation sites. Therefore, the dominant bacterial communities changed in different plots, which was beneficial to the adaptation of microorganisms to the soil environment, could accelerate the stability of soil ecology in reclaimed sites, and was important for the ecological reconstruction of the mine site.

### 4.3. Interactions between Soil C and N Levels and Bacterial Community Structure in Reclaimed Plots

In some cases, changes in the microbial community may precede those in soil physicochemical properties or plants [52]. In this study, both α-proteobacteria and γ-proteobacteria showed highly significant positive correlations with soil carbon, nitrogen, and phosphorus nutrients (*p* < 0.01), indicating that soil nutrients positively contributed to the relative abundance of α-proteobacteria. In addition, α-proteobacteria and γ-proteobacteria as copiotrophic bacteria [53] could use inorganic nitrogen such as NH_4_^+^-N, NO_2_^−^-N, or NO_3_^−^-N as energy sources [54] and promote the nitrogen cycling process of reclaimed soils. The above results indicate that α-proteobacteria and γ-proteobacteria were sensitive to the changes in soil carbon and nitrogen fractions. They could be used as indicators for regular monitoring and fertility assessment, so as to have a more comprehensive and scientific understanding of the status of reclaimed soils.

In this study, the bacterial abundance of α-proteobacteria, γ-proteobacteria, and Gemmatimonadetes showed highly significant positive correlations with soil CEC and SOC contents (*p* < 0.01). Surfaces of soil organic matter could act as sites for cation exchange [55], and meanwhile, CEC affects the retention capacity of soil nutrients [56]. They could promote the changes in soil bacterial community structure [56] to accelerate the process of ecological restoration of reclaimed soils. Different subgroups of Acidobacteria responded differently to soil pH [57]. In our study, Acidobacteria Gp6 was significantly and positively correlated with soil pH (*p* < 0.05). The relative abundances of Acidobacteria Gp6 and the contents of Ca^2+^ and Mg^2+^ were significantly higher in Plot 3, Plot 5, and Plot 6 (*p* < 0.05), and the relative abundance of Acidobacteria Gp6 was highly significantly and positively correlated with soil Ca^2+^ and Mg^2+^ (*p* < 0.01), which was consistent with the results of Huixian natural wetlands, paddy fields, maize fields, and citrus orchards in karst areas [58,59]. The results indicate that some members of Acidobacteria Gp6 were highly adaptable to the environment with alkaline soil and high contents of calcium, and therefore, the role of Acidobacteria Gp6 in the reclamation process of bauxite mine sites in karst areas deserved further attention. The main reason for the phenomenon may be related to the fact that these bacteria have more calcium channels on their membranes [60], but this speculation needs to be proven by further studies.

### 4.4. Effects of Reclamation on Microbial Network Interactions and Keystone Taxa

The soil bacterial networks were dominated by *Copresence* interactions in all plots, indicating that most bacteria in each plot were in cooperative relationships such as mutualism, commensalism, or co-aggregation [61]. The percentage of *Copresence* edges, the average number of neighbors, and network density in the bacterial networks of the control soil were higher than those in reclaimed plots, probably due to the lower contents of nutrients in the control soil which drove the bacteria to occupy different ecological niches as much as possible to utilize the limited resources. For example, the ability of some members of Actinobacteria and Proteobacteria to decompose complex organic matter into simple compounds provided available carbon sources for some other soil bacteria. Some facultative anaerobic or anaerobic bacteria of Firmicutes could utilize carbon sources through fermentation pathways in deep soil, while aerobic bacteria such as some members of Actinobacteria could utilize organic matter in surface soil. The complementarity in ecological niches increased the degree of interdependence and reduced the competition among bacterial populations, which was an outward expression of bacteria adapting to stressful environments such as limited resources [62], so as to maintain the diversity of the bacterial community, enhance the resistance of the ecosystem to disturbance, and to accelerate the stabilization process of soil microhabitats in reclaimed sites.

Modules can be considered as functional units which tend to cluster together in microbial communities [63]. The increase in modularity in networks may represent the differentiation of microbial communities into finer functional units [64], which localizes the interactions of different bacterial species in the network and therefore diminishes the strength of interactions among microbial populations [61]. In this study, the characteristic path length and modularity in the bacterial network of the control soil were the lowest. The results indicate that, on the one hand, the functional units of soil bacteria were more homogeneous under the deficiency of nutrient resources, and bacteria needed to obtain limited resources through strong collaboration. On the other hand, the bacterial community structure under such topological characteristics was unstable, and external environmental disturbance could rapidly transmit to the whole community [65]; therefore, the structure and functions of the bacterial community changed. With the establishment and growth of reclamation plants, the soil physicochemical properties and the nutrients gradually improved and accumulated. Soil ecological conditions continuously improved to drive the changes in topological characteristics in the bacterial network, as shown by the increase in characteristic path length and modularity and the decrease in the total number of edges, the average number of neighbors, and network density, indicating that reclamation plants could improve the ability of the soil bacterial community to buffer environmental changes, which was a concrete manifestation of the stability of the soil micro-ecosystem.

In this study, the keystone taxa of the soil bacterial network differed among all the plots. Among the keystone taxa of the bacterial network in the control soil, the *Blautia* genus, *Coprococcus* genus, Ruminococcaceae family, and Clostridiaceae family all belonged to the Clostridia class of Firmicutes, of which *Blautia* were obligate anaerobic Gram-positive bacteria, and the rest of the bacteria also mostly existed in anaerobic or dysaerobic environments with a powerful capacity for fermenting carbohydrates. The results show that the relative abundance of Clostridia was highly significantly negatively correlated with TN (*p* < 0.01), indicating that a high content of soil nitrogen may inhibit the growth of Clostridia. The keystone taxa in the reclaimed plots mostly belonged to Proteobacteria, Acidobacteria, Actinobacteria, and Gemmatimonadetes, indicating that the bacterial keystone taxa were constantly changing to adapt to the changes in the external environment to accelerate the stabilization of the bacterial community structure in reclaimed soils. In addition, the relative abundances of some keystone species such as ASV 1065, ASV 806, ASV 1021, and ASV 1802 were low, indicating that keystone species may not need high abundance necessarily, but they could provide greater connectivity to the community to enhance the interactions of bacteria beneficially [66].

### 4.5. The Driving Effects of Reclamation on Potential Functional Changes in Soil Bacteria

The chemoheterotrophy and aerobic chemoheterotrophy bacterial functional groups are widespread soil microbial metabolism types [67]. They both are also dominant functional groups in reclaimed soils, participating in important ecological processes such as soil carbon and nitrogen cycling, and accelerating ecological restoration and fertility recovery. It has been reported that carbon in reclaimed soils was the most important factor affecting the composition and gene abundance of bacterial carbon-sequestering taxa [68]. In this study, the chemoheterotrophy and aerobic chemoheterotrophy functional groups were mainly formed by bacteria of Proteobacteria (50.03%), and the abundance of these two functional groups could range from 35.66% to 48.26%, indicating that bacteria in reclaimed soils mainly obtained energy by oxidizing organic carbon rather than sequestering carbon. Fermentation is an anaerobic pathway of carbohydrate metabolism. In our study, the relative abundance of fermentation bacteria in the control soil was the highest, which was mainly from the most abundant class—Clostridia. Possible reasons for the high abundance of fermentation bacteria were the low organic matter in the control soil itself as well as the rolling and disturbance of the soil by large machinery during site leveling, which led to low levels of soil porosity and dissolved oxygen, limiting the tricarboxylic acid cycle (TCA) process [69]. The above results suggest that reclamation affected the patterns and functions of bacteria to utilize carbon sources.

Nitrification is a central link between nitrogen fixation and denitrification in the soil nitrogen cycle and determines the soil N retention ability [70]. The nitrification functional groups in this study were mainly from the Nitrospirae, β-proteobacteria, and γ-proteobacteria. *Nitrosococcus* of γ-proteobacteria and *Nitrosomonas* of β-proteobacteria are both autotrophic ammonia-oxidizing bacteria [71] and are involved in the process of ammonia oxidation during the nitrogen cycle. NH_4_^+^-N was the first substrate for nitrification [72], and the high NH_4_^+^-N content in Plot 2 planted with soybean promoted the growth of nitrifying bacteria; meanwhile, the content of biological nitrification inhibition (BNI) components in most legume root secretions was low [73], which may explain the significantly highest abundance of nitrifying bacteria in Plot 2. In this study, α-proteobacteria accounted for 95.45% of the functional groups of denitrifying bacteria. Many denitrifying bacteria required organic matter as an electron donor and energy source [74], and the high content of organic matter in Plot 5 could provide enough carbon for the denitrifying process; therefore, the intensity of denitrification increased. The above results indicate that Proteobacteria played an important role in the process of nitrogen cycling in reclaimed sites, and the changes in functional groups were an expression of bacterial adaptation to the reclaimed soil environment.

## 5. Conclusions

In summary, reclamation in bauxite mine sites significantly improved the accumulation of SOC and TN. Meanwhile, reclamation significantly changed soil bacterial community structure, which was embodied in the increase in the relative abundance of α-proteobacteria, the decrease in Firmicutes with the reclamation year increasing, and other changes in the bacterial community. Bacteria tended to strengthen the connections and reduce the competition in order to adapt to stressful environments in bauxite mine sites, while reclamation increased the characteristic path length and modularity, which was beneficial to the stability of the bacterial community. Reclamation changed the dominant bacterial functional taxa. Proteobacteria, especially α-proteobacteria and γ-proteobacteria, were significantly related to the content of nutrients and could play important roles in the process of C and N cycling; their abundance might be important indicators of the progress in the reclamation sites of bauxite mines.

## Figures and Tables

**Table 1:** Summary of the plots.

Plot Types	Plots	Reclamation Year	Vegetation	Management Method	Longitude and Latitude
Control	Plot 1	0	Leveled plot, no planting	None	23°25′46″ N, 107°35′21″ E
Reclaimed	Plot 2	1	*Glycine max*	No artificial intervention after planting	23°25′42″ N, 107°35′23″ E
	Plot 3	4	*Leucaena leucocephala*	No artificial intervention after planting	23°23′21″ N, 107°29′30″ E
	Plot 4	7	*Neyraudia reynaudiana*	No artificial intervention after planting	23°23′13″ N, 107°29′48″ E
	Plot 5	10	*Zea mays*	Inorganic compound fertilizers mainly, better human management	23°22′26″ N, 107°30′21″ E
	Plot 6	12	*Brassica pekinensis*	Organic–inorganic fertilizer dispensing, fine human management	23°23′9″ N, 107°30′46″ E

**Table 2:** Basic physicochemical properties of soil in different plots.

Properties	Plot 1	Plot 2	Plot 3	Plot 4	Plot 5	Plot 6
pH (H_2_O)	7.86 ± 0.04 b	5.95 ± 0.03 f	8.06 ± 0.03 a	6.53 ± 0.02 e	7.27 ± 0.02 c	6.74 ± 0.04 d
SOC (g·kg^−1^)	4.22 ± 0.03 e	6.85 ± 0.11 cd	6.50 ± 0.03 d	7.87 ± 0.58 c	19.31 ± 0.68 b	33.59 ± 1.61 a
DOC (mg·kg^−1^)	11.58 ± 0.70 e	14.42 ± 1.14 d	16.88 ± 1.16 cd	18.90 ± 1.66 c	95.96 ± 4.99 b	119.77 ± 4.76 a
TN (g·kg^−1^)	1.37 ± 0.21 e	2.77 ± 0.15 c	2.30 ± 0.10 d	2.43 ± 0.21 cd	3.47 ± 0.25 b	4.57 ± 0.23 a
AN (mg·kg^−1^)	13.92 ± 0.74 e	32.02 ± 0.75 c	32.71 ± 0.44 c	20.20 ± 1.26 d	52.86 ± 1.07 b	78.10 ± 1.98 a
NH_4_^+^-N (mg·kg^−1^)	2.48 ± 0.37 d	6.79 ± 0.84 b	4.32 ± 0.49 c	4.23 ± 0.34 c	6.76 ± 0.41 b	19.18 ± 1.18 a
NO_3_^−^-N (mg·kg^−1^)	1.33 ± 0.11 d	1.34 ± 0.08 d	3.04 ± 0.30 c	0.47 ± 0.14 e	4.94 ± 0.25 b	6.00 ± 0.37 a
TP (g·kg^−1^)	0.09 ± 0.01 d	0.12 ± 0.01 d	0.12 ± 0.01 d	0.20 ± 0.04 c	0.58 ± 0.01 b	3.65 ± 0.09 a
AP (mg·kg^−1^)	3.73 ± 0.54 e	5.11 ± 0.73 d	3.10 ± 0.44 e	9.58 ± 0.90 c	27.99 ± 0.62 b	85.70 ± 0.49 a
CEC (cmol^+^·kg^−1^)	3.52 ± 0.75 c	2.65 ± 0.09 cd	4.60 ± 0.13 b	1.92 ± 0.36 d	4.80 ± 0.44 ab	5.55 ± 0.76 a
Ca^2+^ (cmol·kg^−1^)	2.55 ± 0.09 d	1.91 ± 0.03 e	7.68 ± 0.10 a	1.75 ± 0.03 e	3.17 ± 0.05 c	6.18 ± 0.19 b
Mg^2+^ (cmol·kg^−1^)	0.06 ± 0.00 e	0.18 ± 0.00 c	0.15 ± 0.00 d	0.15 ± 0.00 d	0.50 ± 0.01 a	0.23 ± 0.01 b

Note: Different letters denote significant differences; *p* < 0.05; a > b > c > d.

**Table 3:** Soil bacterial α-diversity indices in different plots.

Sample Plots	Chao1	ACE	Shannon	Simpson
Plot 1	1786.03 ± 274.10 ab	1786.23 ± 274.26 ab	6.54 ± 0.43 b	0.996 ± 0.003 b
Plot 2	1658.67 ± 136.21 b	1658.73 ± 136.09 b	6.56 ± 0.10 b	0.996 ± 0.000 b
Plot 3	1907.00 ± 40.71 ab	1907.13 ± 40.64 ab	6.88 ± 0.06 ab	0.998 ± 0.000 a
Plot 4	1959.00 ± 129.55 a	1959.00 ± 129.55 a	6.84 ± 0.07 ab	0.997 ± 0.000 ab
Plot 5	1865.34 ± 41.01 ab	1865.60 ± 40.97 ab	6.98 ± 0.05 a	0.998 ± 0.000 a
Plot 6	1920.67 ± 68.96 ab	1920.82 ± 68.82 ab	6.94 ± 0.04 a	0.998 ± 0.000 a

Note: Different letters denote significant differences; *p* < 0.05; a > b > c > d.

**Table 4:** Key topological characteristics of soil bacteria networks in different plots.

Properties	Plot 1	Plot 2	Plot 3	Plot 4	Plot 5	Plot 6
Nodes	184	214	228	195	218	208
Edges ^1^	2259 (58.39%)	1911 (51.70%)	1968 (51.68%)	1851 (51.92%)	1842 (50.81%)	1773 (52.00%)
Modularity	0.50	0.77	0.74	0.72	0.77	0.76
Characteristic Path Length	3.29	6.95	6.49	6.81	8.66	6.67
Average Number of Neighbors	24.81	17.86	17.26	18.99	16.90	17.05
Clustering Coefficient	0.67	0.75	0.75	0.76	0.76	0.76
Network Density	0.14	0.08	0.08	0.10	0.08	0.08
Diameter of Network	13	22	15	21	26	14

^1^ The content of the parentheses indicated the percentage of *Copresence* correlated edges.

**Table 5:** Keystone taxa of soil bacteria in different plots.

Plots	ASV Id	Closeness Centrality	Betweenness Centrality	Degree	Categories
Plot 1	ASV 49	0.45	0.07	65	*Blautia*
	ASV 152	0.44	0.07	65	*Bacteroides*
	ASV 224	0.44	0.08	64	Ruminococcaceae
	ASV 215	0.44	0.08	60	Clostridiaceae
	ASV 248	0.39	0.02	60	*Coprococcus*
Plot 2	ASV 149	0.19	0.01	29	Rhodospirillaceae
	ASV 609	0.18	0.00	28	Syntrophobacteraceae
	ASV 431	0.19	0.01	27	Myxococcaceae
	ASV 1065	0.18	0.00	27	Solibacterales
	ASV 127	0.18	0.00	27	Acidimicrobiales EB1017
Plot 3	ASV 806	0.19	0.02	30	Bacteria WS3
	ASV 21	0.18	0.01	30	*Clostridium*
	ASV 161	0.18	0.01	30	Acidimicrobiales
	ASV 23	0.18	0.01	29	Peptostreptococcaceae
	ASV 463	0.20	0.06	28	Bacteria WS3
Plot 4	ASV 40	0.20	0.09	38	Peptostreptococcaceae
	ASV 1802	0.20	0.03	38	Acidobacteria
	ASV 124	0.20	0.03	38	β-proteobacteria
	ASV 183	0.20	0.04	37	*Hyphomicrobium*
	ASV 529	0.20	0.03	37	*Kibdelosporangium*
Plot 5	ASV 211	0.14	0.03	30	Proteobacteria
	ASV 164	0.14	0.02	28	Gemmatimonadetes
	ASV 72	0.14	0.02	27	Gemmatimonadetes
	ASV 469	0.14	0.02	26	Chloracidobacteria PK29
	ASV 616	0.14	0.00	25	Acidobacteria Gp6 iii1-15
Plot 6	ASV 149	0.17	0.06	30	Rhodospirillaceae
	ASV 546	0.18	0.03	29	Hyphomonadaceae
	ASV 1021	0.18	0.05	28	β-proteobacteria
	ASV 174	0.17	0.03	27	Acidobacteria Gp6 iii1-15
	ASV 514	0.17	0.01	27	Gemmatimonadetes Gp5

## Supplementary Materials

The following supporting information can be downloaded at: https://www.mdpi.com/article/10.3390/ijerph192416921/s1, Table S1: The Second National Soil Census Nutrient Classification Standard of China, Figure S1: Bacterial community composition at the level of family in different plots, Figure S2: Correlations of dominant family and soil physicochemical properties.
