# Supplementary material for: Effects of Land Reclamation on Soil Bacterial Community and Potential Functions in Bauxite Mining Area
Source: Int J Environ Res Public Health. 2022 Dec 16;19(24):16921. doi: 10.3390/ijerph192416921 (PMC9778865; doi:10.3390/ijerph192416921)
Supplement: Supplementary file 1 [file ijerph-19-16921-s001.zip › ijerph-2013594-supplementary.pdf]

Supplementary Materials:

Table S1. The Second National Soil Census Nutrient Classification Standard of China.

Grades	SOC（$g·kg^{-1}$）	TN（$g·kg^{-1}$）	AN（$mg·kg^{-1}$）	TP（$g·kg^{-1}$）	AP（$mg·kg^{-1}$）	Interpretation
I	>40	>2	>150	>2	>40	Very high
II	30~40	1.5~2	120~150	1.5~2	20~40	High
III	20~30	1~1.5	90~120	1~1.5	10~20	Upper
IV	10~20	0.75~1	60~90	0.75~1	5~10	Middle
V	6~10	0.5~0.75	30~60	0.5~0.75	3~5	Low
VI	≤6	≤0.5	≤30	≤0.5	≤3	Very low

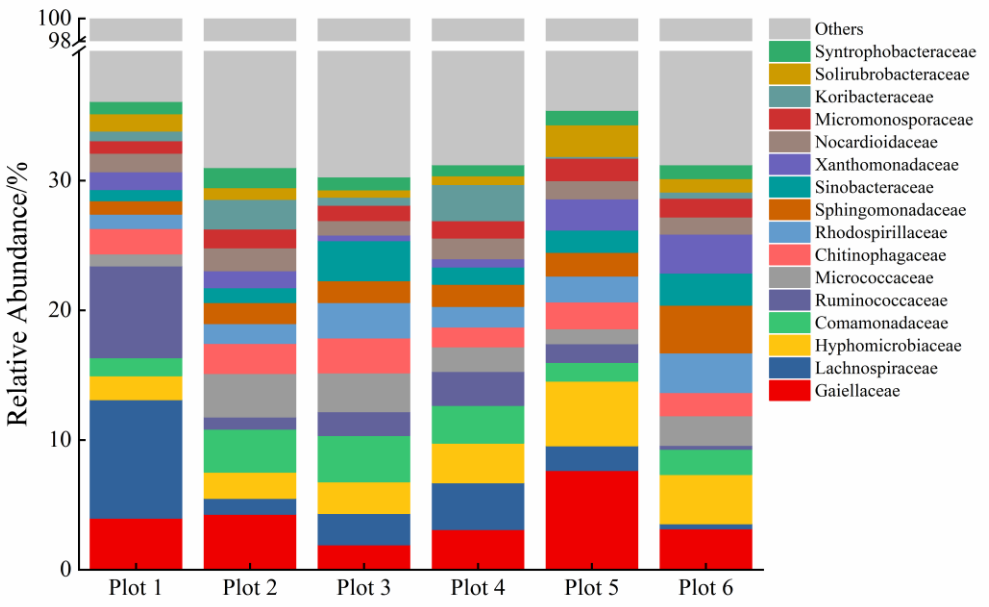

Figure S1. Bacterial community composition at the level of family in different plots. The family with relative abundance greater than 1% were defined as dominant family, and sequences not classified to any taxonomy and phylogenetic groups accounting for ≤1% of all classified sequences are summarized in the group “Others".

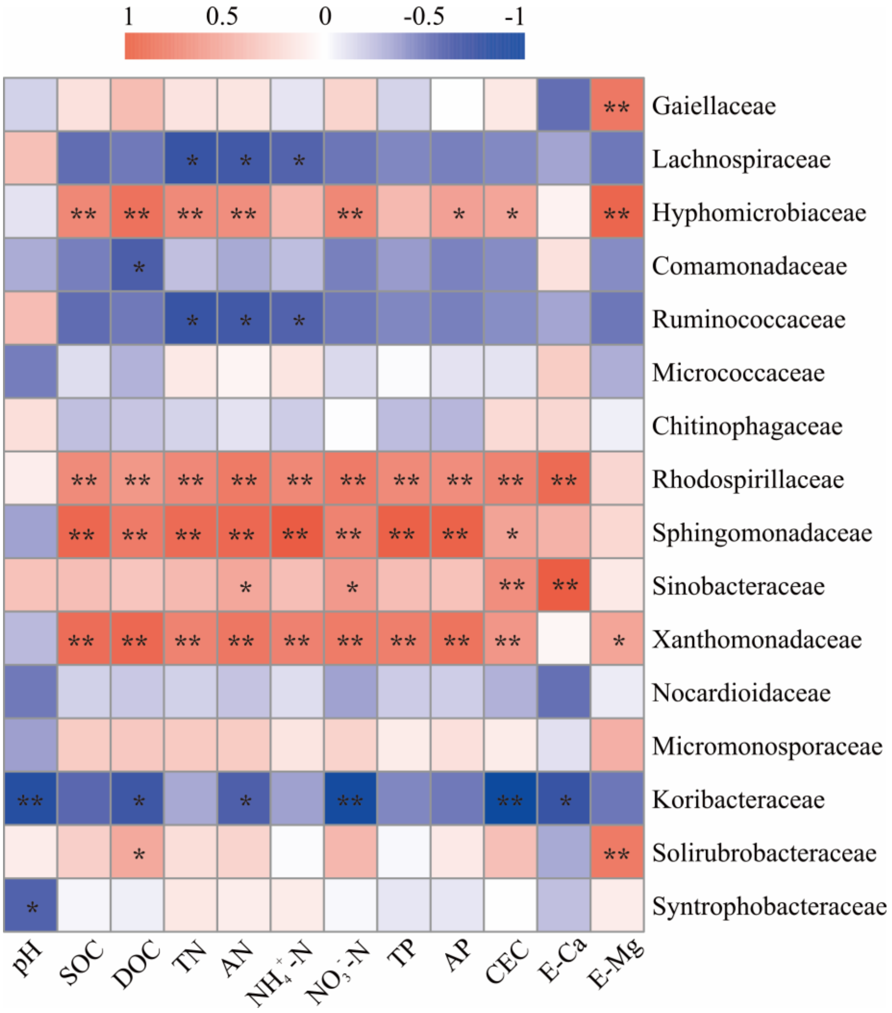

Figure S2. Correlations of dominant family and soil physicochemical properties, * $p < 0.05$ (Pearson correlation, two-tailed), ** $p < 0.01$ (Pearson correlation, two-tailed).
